# Microfocus computed tomography for fetal postmortem imaging: an overview

**DOI:** 10.1007/s00247-022-05517-1

**Published:** 2022-09-28

**Authors:** Daniël Docter, Yousif Dawood, Karl Jacobs, Jaco Hagoort, Roelof-Jan Oostra, Maurice J. B. van den Hoff, Owen J. Arthurs, Bernadette S. de Bakker

**Affiliations:** 1 Department of Medical Biology, Amsterdam UMC at University of Amsterdam, Amsterdam, The Netherlands; 2 Department of Obstetrics and Gynecology, Amsterdam UMC at University of Amsterdam, Meibergdreef 9, Amsterdam, The Netherlands; 3 Amsterdam Reproduction and Development Research Institute, Amsterdam, The Netherlands; 4 Department of Oral Pain and Dysfunction, Functional Anatomy, Academic Centre for Dentistry Amsterdam (ACTA), University of Amsterdam and VU University Amsterdam, Amsterdam, The Netherlands; 5 Department of Radiology, Great Ormond Street Hospital for Children NHS Foundation Trust, London, UK; 6 National Institute for Health Research, Great Ormond Street Hospital Biomedical Research Center, London, UK; 7 Department of Pediatric Surgery, Erasmus MC – Sophia Children’s Hospital, University Medical Center Rotterdam, Rotterdam, The Netherlands

**Keywords:** Anatomy, Autopsy, Diffusible iodine-based contrast-enhanced computed tomography, Fetus, Human, Intrauterine fetal demise, Microfocus computed tomography, Postmortem, Stillbirth

## Abstract

Over the last few years, fetal postmortem microfocus computed tomography (micro-CT) imaging has increased in popularity for both diagnostic and research purposes. Micro-CT imaging could be a substitute for autopsy, particularly in very early gestation fetuses for whom autopsy can be technically challenging and is often unaccepted by parents. This article provides an overview of the latest research in fetal postmortem micro-CT imaging with a focus on diagnostic accuracy, endovascular staining approaches, placental studies and the reversibility of staining. It also discusses new methods that could prove helpful for micro-CT of larger fetuses. While more research is needed, contrast-enhanced micro-CT has the potential to become a suitable alternative to fetal autopsy. Further research using this novel imaging tool could yield wider applications, such as its practise in imaging rare museum specimens.

## Introduction

Fetal autopsy is the modality of choice for diagnosing causes of stillbirth and intrauterine fetal demise (IUFD) and for confirming congenital anomalies. It offers clinically significant findings in approximately 40–70% of cases [1]. Knowing the cause of their loss provides consolation for bereaved parents and offers important information for management of future pregnancies [1, 2]. Over the last years parental consent for autopsy has dropped [1] because parents often view the procedure as too invasive. It is also technically difficult in fetuses at early gestation [2]. To overcome these issues, new and less invasive modalities are being actively proposed.

A promising alternative to autopsy is microfocus computed tomography (micro-CT) [3, 4], a technique that has already made its mark in non-medical industries, e.g., non-destructive precision engineering, ecology and geosciences [5]. Like conventional CT, it is an X-ray-based technology, but instead of a rotating gantry, micro-CT scanners have a fixed radiation source, while the samples are mounted on a rotating platform. The radiation-source-to-sample distance, as well as sample-to-detector distance, can be altered to achieve much higher resolutions, up to sub-micron (<µm) level. In comparison, conventional CT scanners typically have maximum resolutions of 500–1,000 μm. Scan time and radiation dose are typically much higher in micro-CT, clearly making it less suited to imaging live patients, but ideally suited to imaging postmortem fetuses and specimens.

Before imaging, the fetus is treated with staining agents to allow visualization of soft tissues, which otherwise offer very little contrast. Staining is frequently done using iodine compounds as a contrast agent, usually by submerging the fetus in the solution. This technique is referred to as diffusible iodine-based contrast-enhanced CT (diceCT) [6]. Because staining time is directly related to diffusion speed, it takes longer in larger fetuses, ranging from hours to several weeks [5]. Because this method is non-destructive, the fetus can be returned to the parents as soon as scanning is complete or after the discoloration caused by the iodine has been reversed.

Imaging is preferred by parents as an alternative to autopsy because imaging is less invasive and does not leave disfiguration [7]. The opportunities for fetal imaging and research provided by micro-CT imaging have recently been reviewed [8]. Advances have been made with extensive scan and staining protocols [9], such as the use of buffered Lugol solution (B-Lugol), which limits the extent of tissue shrinkage when staining fetuses [10].

In this review we provide an overview of the latest research concerning micro-CT imaging of human fetuses, with a special emphasis on diagnostic accuracy, endovascular staining approaches, placental studies and the reversibility of staining. We discuss new methods that could prove suitable for larger fetuses as well as other relevant techniques that might help to forward fetal postmortem imaging. This review covers data that were previously published, so no ethical approval was required.

## Diagnostic accuracy of microfocus computed tomography

The current reference standard for diagnosis in a postmortem setting is invasive autopsy. Through dissection and microscopic analyses, organs can be visualized to study the anatomy and pathology and to establish the cause of death. Recent studies comparing fetal micro-CT imaging to autopsy found 93% concordance for overall diagnosis among more than 250 cases; in only 1% of these cases did fetal autopsy provide a diagnosis that could not be established using micro-CT scanning [11, 12]. These findings led to a workflow in which micro-CT scanning was used to triage before autopsy, potentially avoiding autopsy in 87% of cases. Concordance between autopsy and micro-CT was met in most cases, with concordance of 97% across all body systems [12]. The largest discordance was found in evaluating the cardiovascular system, which had a sensitivity of 67%; however, this number might be subject to bias because autopsy was only performed in cases where it was expected to add value [12]. In other studies where the heart was extracted before imaging, the sensitivity of micro-CT in cardiac diagnosis has been higher (85–100%) [11, 13, 14].

**Fig. 1:**
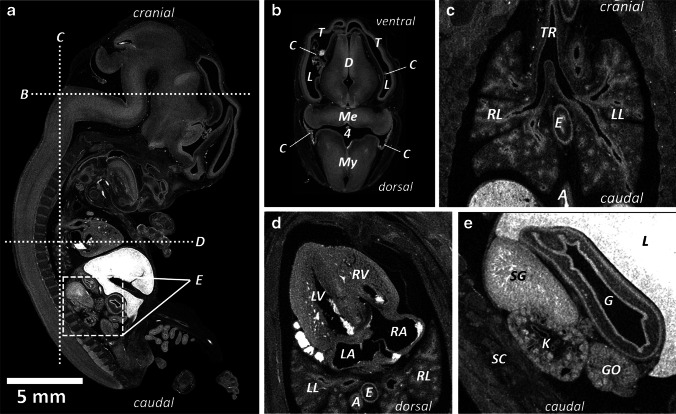
Human embryo at 9 weeks 2 days of gestation, crown-to-rump length of 25 mm, stained by submersion for 90 h in 1.9% buffered Lugol solution (B-Lugol). Microfocus CT scan was obtained using a Phoenix Nanotom M (Waygate Technologies, Wunstorf, Germany). Scan parameters were 60 kV, 400 µA, 0.5-mm aluminum filter, 8.5-µm voxel size. **a** Sagittal image shows an overview of the anatomy, indicating the localization of the images in panels (**b–e**). **b** Transverse coronal image through the brain shows the parts of the neural tube, ventricles and choroid plexus. *4* fourth ventricle, *C* choroid plexus, *D* diencephalon, *L* lateral ventricle, *Me* metencephalon, *My* myelencephalon, *T* telencephalon. **c** Coronal image through the trachea and lungs. *A* aorta, *E* esophagus, *LL* left lung, *RL* right lung, *Tr* trachea. **d** Transverse image through the heart. *LA* left atrium, *LV* left ventricle, *RA* right atrium, *RV* right ventricle. **e** Sagittal image through the region of the right kidney. *G* gut, *Go* gonad, *K* kidney, *SC* spinal column, *SG* suprarenal gland

In fetuses younger than 16 weeks of gestation and in small or macerated fetuses, autopsy can sometimes be difficult or even impossible because of the technical challenges. For example, the fragile and semifluid fetal brain is generally removed from the skull by submersion in a water bath [15] but remains hard to evaluate. Micro-CT imaging, however, can even be used in embryos (Fig. 1) before 8 weeks of gestation [16] and in macerated fetuses [17]. Although at this early stage of gestation, not much is known about pathophysiology because there is a lack of data on these early stages, micro-CT has been found to be a good, if not better, alternative to autopsy for postmortem imaging of fetuses at early gestation [11–13, 16].

To ascertain a diagnosis, histology can be of added value. However, staining with iodine or other solutions and micro-CT scanning might hamper further histological analysis because of the potential disturbances in tissue integrity. Lupariello et al. [18] used cardiac samples to determine the effects of staining and scanning samples prior to histology. Samples were divided into two groups, either stained with a combination of Lugol solution, methanol and Tween (Thermo Scientific, Waltham, MA) or perfused with Microfil (Flow Tech Inc., S Windsor, CT), an endovascular casting agent. After micro-CT scanning, samples were stained with immunohistochemistry, hematoxylin or Masson trichrome. All samples demonstrated histological staining in all tissues, and the Microfil samples showed a brown substance covering the endothelial layer of the arteries [18]. Although the authors did not find that staining hampered further microscopic evaluation of the tissue, the micro-CT scanning itself appeared to alter proteins in these cardiac samples, possibly the result of thermal damage caused by scanning [18]. The extent to which other proteins are denatured or affected remains unknown. This raises the questions of whether deoxyribonucleic acid (DNA) degradation occurs during micro-CT scanning, which needs be evaluated in future studies. One might consider taking microbiopsies for microscopic evaluation or DNA analysis prior to scanning to prevent iatrogenic thermal alteration of the sample, the extent of which is unknown.

## Staining methods

### Whole-body staining by submersion

**Fig. 2:**
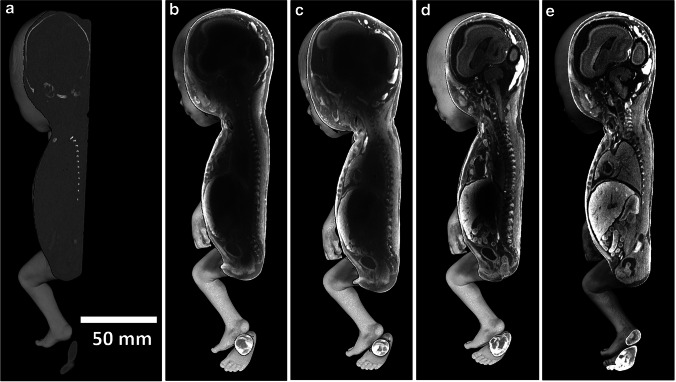
Volume rendering with a sagittal image of a postmortem 19-weeks-6-days fetus, demonstrating staining over time. Staining was achieved with submersion in 7.5% Lugol–formalin solution [9]. Crown-to-rump length is 150 mm. CT images were obtained using a conventional medical scanner (Somatom Force; Siemens Healthineers, Erlangen, Germany). **a–e** Unstained image **a** and images with 25 h of staining **b**, 45 h of staining **c**, 119 h of staining **d** and 241 h of staining **e**

To enhance soft-tissue differentiation, staining is needed before scanning. For human fetuses, the most common method to enhance soft-tissue contrast is staining by submerging the fetus in a staining solution [8, 9]. Frequently used staining agents are based on iodine, phosphotungstic acid (PTA), phosphomolybdic acid (PMA) or osmium tetroxide [6, 19, 20]. For human fetuses, the most frequently used staining solution is a water-based solution containing two parts potassium iodide (KI) for every one part iodine (I^2^), or potassium triiodide (I^2^KI), also called Lugol solution. This solution is known for its rapid and deep penetration, provision of excellent contrast in all tissues and for being non-toxic and relatively cheap, all of which make it a versatile and robust staining agent. Nonetheless, successful staining by submersion in I^2^KI is dependent on several factors, including the size of the fetus, the concentration of the staining solution and the staining time. As fetuses become larger with increasing age, the triiodide must penetrate deeper, requiring longer incubation periods to reach adequate staining of all structures (Fig. 2) [9]. Moreover, with increasing gestational age the skin of the fetus becomes less penetrable to iodine, prolonging the staining time. Staining concentration and time are interdependent factors in the staining process. A shorter staining time can be reached with higher concentrations because faster diffusion occurs, although higher concentrations can result in overstaining, causing loss of tissue differentiation [6].

Although some institutions have used unbuffered Lugol solution for fetal staining without encountering significant tissue shrinkage [9], users should be aware that Lugol solution can cause tissue shrinkage of up to 30% [8, 10, 21]. This negative effect on the tissue was long thought to be the result of an osmotic imbalance between the staining solution and the fetus; however, the use of an isotonic staining solution did not prevent tissue shrinkage, thus demonstrating that osmotic imbalance is not the driving factor. Dawood et al. [21] demonstrated that acidification of the solution is the key factor in soft-tissue shrinkage rather than the osmolarity of the staining solution [10]. Moreover, the authors found that staining in Lugol solution prepared with a buffer (B-Lugol) stabilizes the pH and almost completely prevents soft-tissue shrinkage, without affecting the staining time [10].

### Endovascular diffusion staining

Whole-body staining by submersion has proved effective for excised tissues [22] and small whole-body fetuses (< 20 weeks of gestation). However, as mentioned, complete penetration of the staining agent takes longer when there is increased distance between surface and center of the fetus and requires large volumes of submersion fluid in bigger, i.e. older fetuses. Moreover, the increased skin density and ossified structures (e.g., the skull) may form barriers and hamper diffusion in older fetuses. This is particularly evident when aiming at complete staining of the brain in older fetuses in cases where the skull is formed. Depending on the size of the fetus, staining time ranges from a couple of days to several weeks. When providing a postmortem scanning service as an alternative for conventional autopsy, this delay is undesirable.

Endovascular infusion of the contrast agent offers a possible alternative for staining by submersion to accelerate uptake and direct delivery to peripheral tissues. In theory, this would create a larger surface area and bypass hard-to-penetrate structures such as the skin and skull. To the best of our knowledge, this technique has not been used for whole-body staining of human fetuses to date. However, it has been tried in animal experiments using mice [23–25] and newborn piglets [26]. In mice, contrast agents were either administered into the left ventricle of the heart [23] or retrograde through the aorta [24]. Before administration of the contrast agent, the mice were heparinized to prevent blood clotting [23, 24]. After infusing the staining agent, the fetuses were fixed [23]. Staining agents PTA and Lugol were tested, and Lugol was found to have a faster uptake (15 min vs. 30 min stain time) and showed more homogeneous staining throughout the body compared to PTA. This may be because triiodide is a smaller molecule than PTA. Lugol also resulted in better contrast in the myocardium and bronchial walls than PTA. On the other hand, PTA appeared to have more uptake in the liver, renal parenchyma and vessel walls. So, if the aim of the staining is to visualize the vasculature, then PTA would be the preferred contrast agent [24].

Schweitzer and coworkers [26] recently presented the first results using diluted barium sulfate solution in combination with a high-pressure (max. 1.4 bar, max. 22 L/min) angiography pump for venous and arterial filling in postmortem newborn piglets. This procedure results in staining of the entire fetus without macroscopically visible discoloration of the tissues [26]. In their studies, adequate staining could be achieved even several days after the animals’ demise while they were stored in a cool environment. These studies approach the needs of a clinical setting with delayed staining, for example if parents wanted some time with their child before diagnostics or there were logistical delays to staining. Time between demise and staining, however, should be kept short to prevent too much clotting of the blood and decay of the body.

Only one study reported on the comparison and combined administration of Lugol through submersion and endovascular infusion. Fixed mice were either injected with Lugol in the right ventricle or submerged in Lugol after their skin was incised, in both cases using a similar concentration of Lugol solution; the endovascular approach failed to deliver contrast because the staining agent remained within the heart and was not distributed to the body [25]. This is likely the consequence of specimen fixation prior to infusion of the agent and the lack of perfusion with heparin to prevent clotting.

Endovascular delivery should be further explored because it might dramatically reduce staining time [23, 24]. However, it becomes increasingly technically difficult to administer a staining agent in smaller vessels, and for this reason submersion is likely to remain the staining method of choice for the smallest fetuses. Intravascular administration of various staining agents and concentrations might provide new ways of visualizing anatomy of large fetuses and thereby form a basis for future studies, although any interference with the body including needling or injection might be unacceptable to parents.

## Placental research and intravascular casting agents

The placenta is the chief regulator of nutrient supply for the fetus, and placental disorders such as pre-eclampsia can impact the health of both mother and fetus. Pathogenesis of pre-eclampsia is not fully understood, and by visualizing the smallest vasculature of the placenta, new light could be shed on this problem and advance our understanding of such diseases. Two umbilical arteries and one umbilical vein provide the blood circulation of the fetus. They branch out in the placenta to form a complex network that is anatomically variable and is, even in healthy placentas, not fully understood. It has been investigated using micro-CT [27, 28]. For intravascular and placental research, Microfil and BriteVu (Scarlet Imaging, Murray, UT) are two frequently used casting agents [27, 28]. These agents are infused into capillaries while in liquid form and are allowed to solidify into a highly dense intravascular cast that can be visualized using various medical imaging techniques, micro-CT among them. The contrast agent does not leave the vessel and does not cause staining of soft tissue and therefore provides visualization exclusively of the vascular anatomy [29]. In general, care must be taken that the tissue is not perfused with a pressure higher than the physiological blood pressure because this leads to damage of the vessels and overestimation of vascular volume from vessel expansion.

Microfil is a lead-based substance designed for visualizing microcirculation of vessels larger than 100 microns [30]. Before administration, the vessels must be flushed with sodium chloride and perfused with heparin to prevent clotting for maximal perfusion of the substance. Aughwane and coworkers [27] infused Microfil into term placentas and found that only half of the vessels larger than 200 µm^2^ were perfused adequately (more than 75% filling), which might indicate that Microfil is less suitable than anticipated to visualize the microcirculation.

**Fig. 3:**
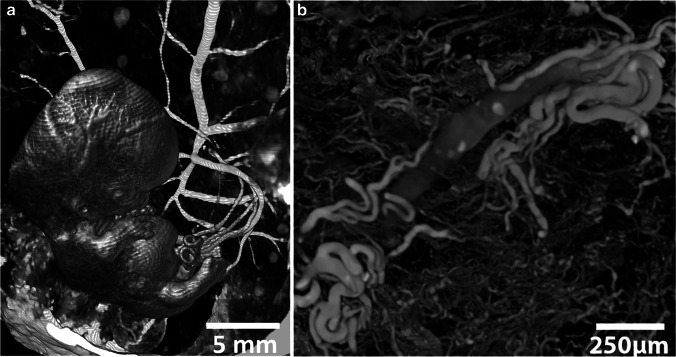
Microfocus CT (micro-CT) scans of feto-placental vasculature. **a** Micro-CT volume rendering of intact pregnancy terminated at 6 + 6 weeks post conception. Crown-to-rump length is 22 mm. Scan was derived from the Dutch Fetal Biobank. **b** Micro-CT volume rendering of a subsample taken from a growth-restricted placenta infused with BriteVu demonstrates an example of unexpected branching characteristics observed at the level of the meso-vasculature. Image adapted from James et al. [28] with permission

BriteVu is a barium sulfate–based substance that is less viscous than Microfil [28]. Using BriteVu, James and coworkers [28] were able to visualize capillary vessels down to 10 μm wide. From their study, it is not clear whether BriteVu reached the vasculature through the entire fetus because only specific areas are shown. In detailed images (Fig. 3), the vessels appear to be well perfused, supporting their findings, and one might anticipate that the entire vasculature of the fetus can be reached [28].

These casting agents have been used to study the microvasculature of the placenta, but no researchers have been able to scan a complete placenta at the resolution needed to image the 10-µm wide micro-vessels [28]. For vascular research it remains questionable whether it is necessary to visualize the smallest vessels, considering their anatomical variability. Depending on the expected size of the studied entity, intravascular casting agents could be an asset in future studies. Note, however, that endovascular casts might interfere with (subsequent) soft-tissue staining, especially in endovascular approaches.

## Reversibility of staining

**Fig. 4:**
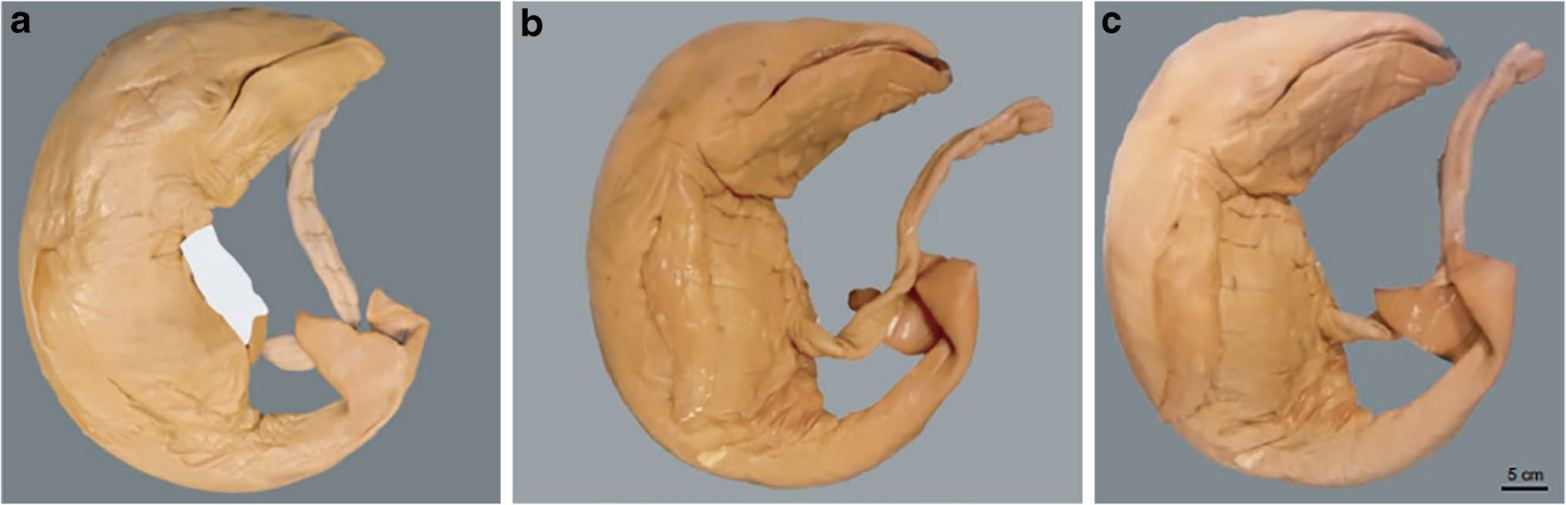
Humpback whale fetus before and after staining and de-staining in lateral view. **a** Initial state stored in 70% ethanol. The white area on the anterior side covers the specimen’s label. The label was removed to avoid damaging it in the staining process. **b** Specimen after 3 weeks of staining with a solution of 1% metal iodine in ethanol. **c** Result after 2 weeks of de-staining using a solution of 3% sodium thiosulfate. Adapted from Lanzetti et al. [31] with permission

After iodine staining of fetuses, there is potential skin discoloration to a brown color, which might be undesirable to parents if they are not forewarned that this can happen, or if scanning is done on rare museum specimens as an alternative for the inevitable destructive effects of dissection. Lanzetti and coworkers [31] found that in animal museum specimens that were preserved in 70% ethanol and stained using 1% iodine in 70% ethanol, the discoloration could be reversed using 3% sodium thiosulfate (STS) in 70% ethanol within hours to a few days (depending on fetal size) [31]. Figure 4 shows an example of a very rare humpback whale embryo that was stained and de-stained according to this protocol.

Although specimens are de-stained on the outside, they might still be stained internally [31]. When scanning postmortem human fetuses, superficial de-staining might be sufficient for parents if this accelerates the process of returning the body to the family. For rare museum specimens this might not be sufficient and requires future studies. Moreover, it should be noted that STS does not restore a fetus to its earlier chemical state because STS reacts with the brownish triiodide and reduces it to the colorless iodide. To remove the iodide from the iodine stained specimen, further incubation steps are required, though it should be considered that these additional incubation steps might alter the fetus because of an osmotic imbalance between the incubation solution and the tissue. This process, called leaching, may easily take several weeks or months, depending on the size and volume of the fetus, and requires regular handling to refresh the solution as it saturates with iodide [8]. Longer de-staining times are, however, less of an issue in the setting of museum specimens. The notion that ethanol-preserved specimens can be stained, scanned and de-stained without causing damage might provide an opportunity to scan even rare pathological human fetal specimens and thus give more insight in human fetal development by utilizing rare collections.

## Conclusion

Over the last decade a considerable number of studies have been published regarding micro-CT imaging of human fetuses. The use of micro-CT offers high diagnostic accuracy in the clinical setting comparable to that of a classic autopsy. It should be noted that even after autopsy, 55% of intrauterine fetal demise cases are still left unexplained [11, 12], but this information can help counsel parents appropriately following pregnancy loss. It is to be expected that the diagnostic accuracy of fetal micro-CT imaging will increase in the coming years because of advancing techniques and protocols, improved scan quality and the growing experience of developmental biologists, radiologists and radiographers.

With further research, we anticipate that contrast-enhanced micro-CT will become a suitable alternative to fetal autopsy at early gestation and for rare museum specimens. Moreover, the acquired micro-CT images can be made available worldwide to be studied and rendered into three-dimensional models for both research and wider teaching.
